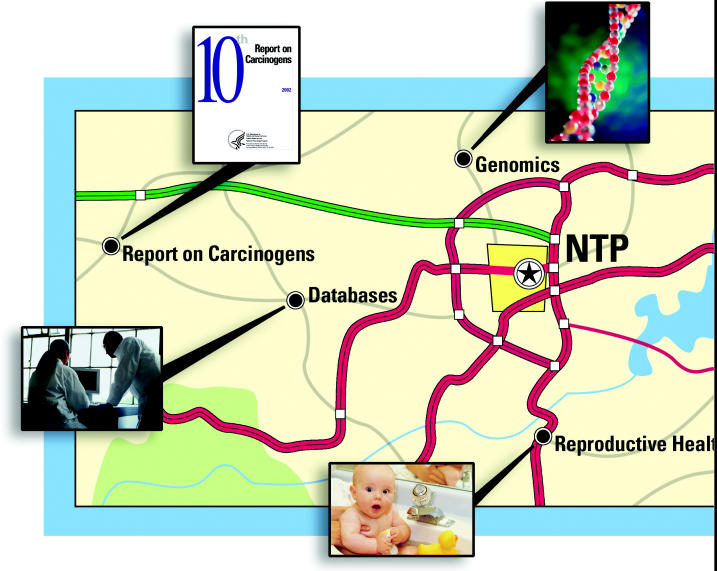# National Toxicology Program: Landmarks and the Road Ahead

**DOI:** 10.1289/ehp.112-a874

**Published:** 2004-11

**Authors:** Victoria McGovern

The National Toxicology Program (NTP), a cross-agency unit of the Department of Health and Human Services (DHHS), is one of the focal points for government efforts aimed at generating, collecting, and coordinating data used for guiding public health decisions. Now 25 years old, the NTP is in the middle of a strategic planning effort to define how it will integrate new technologies with classical toxicological approaches to continue providing, according to the NTP’s motto, “good science for good decisions.”

In its current form, the NTP integrates activities from the NIEHS, the National Institute for Occupational Safety and Health (NIOSH), and the Food and Drug Administration (FDA) National Center for Toxicological Research (NCTR). Its mission is to evaluate agents of public health concern by developing and applying tools of modern toxicology and molecular biology, a mission it achieves through the use of several strategies. It designs studies on potential toxicants and works with outside groups and government labs to carry them out. It reviews and evaluates what’s missing in understanding environmentally induced diseases. It then seeks to fill those gaps by collaborating and cooperating with federal agencies and with other domestic and foreign toxicology and public health organizations, and by carrying out research itself on human exposure and toxicity at laboratories housed at NIOSH and the NIEHS. It supports grants, contracts, and interagency agreements made through the NIEHS Division of Extramural Research and Training, and it supports the activities of three centers.

These activities have brought immense gains in knowledge for the scientific community. But there is more work still to do. Since the fall of 2003, the NTP has been engaged in developing and seeking public comment on a “roadmap” to guide the program’s route forward over the next decade. The roadmap seeks to build on recent technological advances that will allow toxicology to evolve from a largely observational science to one that is more predictive, and thus in many ways more protective of public health.

## The Journey Begins

The NTP’s roots reach to the late 1960s, when concerns over the effects of environmental chemicals on human health began to grow among scientists, policy makers, public health officials, and the general public. Books like *Silent Spring* began to describe the relationship between humankind and the rest of the world as dangerously out of balance. The growth of the environmental movement in this decade led to greater public awareness of natural resources and the potentially harmful effects of environmental pollution. As law makers and the public became increasingly concerned about unforeseen effects of chemicals all around us—particularly those used in manufacturing, agriculture, and household products such as cleaners—new scientific and regulatory approaches were enacted to better understand these agents and decrease or eliminate human exposure to harmful agents.

Through the 1960s and 1970s, new agencies were formed and responsibilities within existing agencies were realigned in efforts to understand, classify, and/or regulate compounds of concern. The Toxicology Information Program at the National Library of Medicine began gathering toxicological information and compiling data banks to allow searching and comparison of the assembled material. The NIEHS, which was established in 1966 and became a full NIH institute in 1969, started a laboratory focused specifically on neurotoxicology in 1977, and in the same year hosted one of the first conferences on environmental estrogens. The Environmental Protection Agency (EPA) was founded in 1970 to pull environmental and health protection activities from across the government into one administrative unit. The FDA took on licensing authority for new biological therapeutic agents but transferred its responsibility for oversight of potentially hazardous materials and other dangers in home items (such as toys, furniture, clothing, and household chemical products) to the Consumer Product Safety Commission (CPSC), which was established in 1972.

In November 1978, Joseph Califano, secretary of the Department of Health, Education, and Welfare (the predecessor of today’s DHHS), established the NTP as a cooperative and coordinating effort between agencies involved in public health. The program was seated at the NIEHS and placed under the leadership of then–NIEHS director David Rall. Kenneth Olden assumed directorship of both the NIEHS and the NTP in 1991. Despite this link, the NTP was and remains independent of the institute and of NIH.

The new program’s charge included coordinating toxicological testing programs within public health agencies; strengthening the scientific basis of toxicology; developing and validating new assays and improved testing methods; and providing information about potentially toxic chemicals to health, regulatory, and research agencies across the government, to the scientific and medical communities, and to the general public.

## Strengthening the Scientific Basis of Toxicology

Among its earliest activities, the NTP began collecting data from work going on across federal agencies; by 1980 it had generated a database comparing the results gained from a variety of widely used genotoxicity assays. By the end of that year, the program had issued its first *Report on Carcinogens* (*ROC*), a scientific and public health document identifying substances, mixtures, and circumstances of exposure that may lead to human cancers. The *ROC* has been updated periodically since then, and the eleventh edition is scheduled for release late in 2004.

In 1983, the NTP first developed and began using five standard categories to summarize the strengths of experimental data produced by its own laboratories and those of other agencies and industry in studies of chemical or physical agents. Four categories are based on a scale of confidence that ranges from “clear evidence” to “no evidence” of harm; a fifth judges that evidence amounts to an “inadequate study.” The standardization helped make the program’s long-term toxicity reports—which can form the basis for policy recommendations on acceptable exposure levels and use of chemicals—more consistent across the range of agents and exposures studied. So, for example, when the State of California passed Proposition 65, the Safe Drinking Water and Toxic Enforcement Act of 1986, NTP data from the *ROC* were available to help the state set standards for discharge of potentially harmful agents into drinking water. By 1987, the NTP’s standardized categorization had been used to classify conclusions from earlier federal studies of potential hazards, allowing the older information to be integrated into current data sets.

In a paper in the 22 May 1987 issue of *Science*, the NTP published the first comprehensive evaluation of genotoxicity assays, laying the groundwork for a more systematic approach to developing and validating new *in vitro* assays. At the same time, the program developed a battery of tests for assessing chemically induced immunotoxicity. As the 1980s drew to a close, the NTP began developing transgenic mouse models for toxicology and carcinogenicity testing, a new approach that would allow easier detection of end points such as tumors. (The NTP has continued to investigate the development of transgenic animals as research tools, and in 2003 launched a new technical report series to convey the findings from transgenic model systems.)

## Revving Up in the 1990s

The 1990s saw the NTP embark on a series of new directions. One new effort, the Predictive-Toxicology Evaluation Project, was launched in 1990. Designed to use chemical structures for direct prediction of bioassay outcomes, the project published its first results in the October 1996 issue of *EHP Supplements*. Another new focus evolved in 1992, when the FDA and the NIEHS formed an interagency agreement to coordinate and jointly fund a phototoxicology research facility at the NCTR in Jefferson, Arkansas. Existing laboratory space at that facility was renovated in 1998 and 1999 to form a dedicated NTP Center for Phototoxicology. The center works to address the carcinogenic potential of chemicals when they are exposed to light or applied to photo-treated skin.

The NIH Revitalization Act of 1993 required that the NIH focus more on developing methods that lessen the use of research animals, reduce pain and distress in animals used, or avoid using animals altogether. In 1997 Olden put together a cross-agency panel, the Interagency Coordinating Committee on the Validation of Alternative Methods (ICCVAM), to implement the act’s requirements. The committee was made permanent in 2000 with the passage of the ICCVAM Authorization Act. The NTP Interagency Center for the Evaluation of Alternative Toxicological Methods was founded in 1998 at the NIEHS to provide support for ICCVAM’s activities by holding workshops and meetings, and by generating reports. Through their efforts, the first alternative test acceptable to the EPA, the FDA, and the Occupational Safety and Health Administration, one for allergic contact dermatitis, was established, significantly decreasing the number of animals tested for this end point.

Through the 1990s, emerging public interests led the NTP in new pursuits. With the passage of the 1994 Dietary Supplement Health and Education Act, the NTP began looking at the potential for toxicity and carcinogenicity of selected dietary supplements, medicinal herbs, and the compounds within them. The *ROC* also saw some changes. The report was put on a biennial publication schedule in 1993. The NTP’s nominations process, by which federal and nongovernmental entities can recommend agents for testing, was made clearer and more open to public scrutiny. In addition, new criteria allowing broader consideration of mechanistic data were added to the *ROC*.

A third NTP center, the Center for the Evaluation of Risks to Human Reproduction, was established at the NIEHS in 1998. The center provides scientifically based uniform assessments of the potential for reproductive and developmental damage from human exposure to certain toxicants. Expert panels convened by the center evaluate evidence of reproductive toxicity, determine patterns of chemical use and human exposure, and develop scientific consensus around tested agents’ capacity to harm humans.

NTP workshops, conferences, and reports in recent years have covered a broad range of emerging and continuing toxicological issues. The program has addressed exposures of concern such as methylmercury, acrylamide, and genetically modified foods. Researchers have developed and validated technical methods for assessing human exposures, performing dermal exposure studies in animals, and detecting endocrine disruptors. And the program has brought researchers together to build scientific consensus on issues including how thyroid hormone affects reproduction, approaches to selecting agents to study in transgenic mouse models, and assessment of acute systemic toxicity.

## Establishing a Roadmap for the Future

In 2003, the NTP began to look at how it could better position its activities to take advantage of new technologies and address a broader range of toxicological end points. “The NTP has been in existence for twenty-five years,” says Christopher Portier, associate director of the program. “It was time to look at what we’re doing and decide if it’s still the right thing.”

In fall 2003, the NTP’s advisory Board of Scientific Counselors (BSC) developed and approved a vision statement: “to support the evolution of toxicology from a predominantly observational science at the level of disease-specific models to a predominantly predictive science focused upon a broad inclusion of target-specific, mechanism-based, biological observations.” Three workgroups—one from the NIEHS, one representing all of the federal agencies served by the NTP, and one that was a subcommittee of the BSC—then contributed comments on what it would take to make the vision a reality. Their comments were incorporated into a revised statement, which the NTP released to the public in late 2003. At the same time, the NTP announced a year-long process to develop and seek public comment on a “roadmap” for achieving the vision.

The NTP’s effort is not a part of, but rather complements, NIH’s recently completed Roadmap for Medical Research. The latter roadmap was aimed at identifying critical gaps and opportunities in biomedical research that do not fit clearly into the mission of any of the member institutes.

The process of developing the NTP roadmap has been very open: the vision and roadmap were discussed at a public meeting at the NIH’s Bethesda campus in January 2004; at a meeting of the NTP’s Scientific Advisory Committee on Alternative Toxicological Methods held in March; at a meeting of leading toxicologists held the same month at the conclusion of the Society of Toxicology’s annual meeting; at the June meeting of the BSC; and at a retreat with advisors, stakeholders, and federal agency staff in August.

Those commenting were asked to address what information the NTP should be producing and what technical capacities it should have at different points over the next 10 years; how refinement and replacement of classical toxicological studies with mechanism-based assays will affect the evaluation of public health hazards; how the NTP can best be structured to provide this information and ensure its optimal utilization in the protection of public health; and what resources and length of time will be needed to realize this vision.

Early on, says Mark Toraason, a NIOSH representative to the interagency committee providing input on the roadmap, the vision focused solely on turning toxicology on its head by moving it from an observational science to a predictive science. But because of perceived limitations, the vision did not get immediate buy-in from all stakeholders. “It was like, ‘Wait a minute, it may be NTP that generates that data, but it’s other government agencies that use it. The other government agencies should be indicating what they need, and NTP should meet those needs,’” says Toraason. “There has been a meeting of the minds there, and the roadmap has been modified with the recognition put in that the needs of the other government agencies can continue to be met as the vision is attained.”

Short-term goals (to be accomplished in the next five years) include a number of workshops to set priorities and working strategies. Basic study design, from choice of model organism strain and species to duration of experiments, will be re-evaluated, and gaps where current study designs or the current knowledge base fall short will be addressed. NTP staff and advisors will interact with policy makers to build better tools for analyzing risks and setting standards. Proof-of-principle studies will be conducted with new and revamped assays, and new data infrastructure and data management tools will be built. Work toward improving the NTP’s use of high-throughput screening will include cataloguing approaches being used in the public domain and applying these assays to more than 500 agents that have already gone through the full two-year bioassay that is the NTP’s current gold standard.

Long-term goals (to be accomplished over the next 10 years) are ambitious—validating the use of new kinds of assays for regulatory decision making, prioritizing compounds for toxicity testing, evaluating human relevance, and addressing specific diseases. In the long run, the NTP aims to develop and validate a battery of predictive tests and a systematic understanding of the metabolism of toxicants. The plan also calls for banking tissue samples from NTP studies and panels of tested agents, which would be available to the extramural research community by 2010.

## Wheels of Change

With rapidly developing technologies creating new opportunities for doing better science faster, application of high-throughput approaches—including whole-genome analysis and work that builds on genomic data—is a critical part of the roadmap. These new approaches show great promise for solving a central problem: the sheer volume of chemicals that must be characterized and tested. Various estimates put the number of chemicals in commerce in the tens of thousands; Portier says such estimates are almost certainly an understatement. “We can’t possibly test everything,” he says. EPA scientist and Society of Toxicology president Linda Birnbaum puts it another way: “We can’t keep doing more and more and more tests on more and more and more chemicals. We have to be able to test smarter, not necessarily more.”

“I think we can double, triple, quadruple, tenfold the number of things we look at if we’re intelligent about how we approach it,” Portier says. He explains using an example from the world of industry: “In developing a new drug, the pharmaceutical industry scans a very large number of chemical entities in some very simple assays. From that, they choose a smaller set that they test in medium-throughput assays, and from that they choose one or two that they test in animals. That’s the direction I want to see us going.”

But moving in new directions isn’t easy, and incorporating new technologies into the NTP’s suite of well-accepted assays provides one of the biggest challenges. “One of the problems with developing totally new methods is when you do something new, it has to be replicated, which means that the expertise and the work have to be going on in a number of different laboratories,” says George Daston, a research fellow at Procter and Gamble and member of the BSC.

Validation of the assays developed around new technologies will be critical. “The technology is quite enticing and it’s razzle-dazzle, but we always have to be able to bring it back to the reality of biology and what it means for the whole organism, and especially what it means for human risk,” says Samuel Cohen, a professor at the University of Nebraska Medical Center in Omaha and BSC member. “This needs to be an evolutionary process rather than a revolutionary process.”

Reduction, refinement, and replacement of animal use may be one of the areas where this evolution first appears. Currently the NTP favors the two-year animal bioassay as its assay of choice. Yet adding more analyses to the experiments currently being done could help, says Cohen: “There is a considerable amount that can be gained, not only from the animals during the two-year process, but also in the preliminary studies—the four-week and thirteen-week studies that are done ahead of time—that could be of more predictive value, so that we didn’t have to do as much in the two-year study ultimately, if at all.”

## All for One and One for All

Data sharing will help bring stakeholders together. The NTP intends to eventually make its chronic assay database and other databases publicly available, which Toraason says will help with communication, not just to the public but also across federal agencies. “In the past it could be difficult to get information from NTP at times,” he explains. “You needed a reliable contact and had to know who knew what and so on. Now their website contains a wealth of searchable information, and the intent is to increase the amount of information that is publicly available.”

The extramural research community will have access to all these public data and can expect to play new roles if the roadmap is put in place, but it’s not yet clear how they will be involved. “We talked about a large number of possibilities,” Portier says, “from providing the extramural community with NTP tissues from NTP studies to do their own research, to providing them with arrays of chemicals if someone has their own high-throughput facility, to setting up centers for doing toxicological screenings toward mechanisms, and a number of other different possibilities.”

The work of extramural scientists will be needed and appreciated. “We don’t have all the answers, we don’t know how to analyze all the data, we don’t know what it all means, and I’m not sure I have the staff on hand to do all that,” Portier says. “The only way it’s going to actually happen is if the public has access to the data. It’s going to be scientists at universities who come out and get NIEHS grants to analyze our data and give us some interpretation tools that really lead that effort.”

James Popp, a BSC member and cofounder of the consulting group Stratoxon, has worked in academe, government, and industry. He says there’s a much greater openness among those three realms to talking and working together than he’s seen in a long time. “There’s been this mind-set change, and I think that’s important to opening up these dialogues,” he says. “We’re in an era of very rapid technological changes, and so it’s a matter of capturing that opportunity of mind-set changes, technology changes, and new advancements in basic knowledge of biology.”

This greater accord between sectors raises some concerns, though, according to Jennifer Sass, a senior scientist for the Natural Resources Defense Council, an environmental advocacy group. “The collegial familiarity that’s being set up leads to a lot of questions,” she says. “The problem is that industry clearly sees a triangle including government, academia, and themselves, and there is no public interest, no public watchdog groups, in the picture.”

Other opinions vary. “NTP has done a good job of informing just about everybody in the scientific community about what they’re doing. They have listened to the criticisms, and have expanded the vision accordingly,” Toraason says.

Still, the public does need to be informed, says Popp, and that will take better efforts at communications. “There are multiple audiences,” he explains. “Obviously, there’s the political audience, but it’s more than that—it’s a broader societal audience that needs to be addressed, and the general public does not have a good grasp of basic scientific principles in toxicology or anything else. So there’s a very large educational process that needs to go along with the scientific work in the roadmap.”

## The Road Ahead

The roadmap sets an ambitious course for the next decade. But is it achievable? “[The NTP has] to recognize that what they put out is a vision, very broad and somewhat all-encompassing,” says Michael Holsapple, executive director of the Health and Environmental Sciences Institute of the International Life Sciences Institute, an industry-funded nonprofit that works to enhance the scientific basis for public health decision making. Holsapple recommends “partnering, being judicious, and recognizing that their map is a good first step.” But in reality, he says, “when the rubber hits the road, they can’t do it all.”

Portier sees it differently. “Partnerships will be an important part of the initiatives outlined in the roadmap,” he says. “While the NTP has the budget necessary to address all of the aspects of the roadmap, it will require a concerted effort from all of the NTP stakeholders to develop the tools that will allow us to understand and use this new information in addressing public health priorities.”

Everything could change with the arrival of a new director. Olden has announced he will leave his positions as NTP and NIEHS director in the near future, though he will continue to serve until his successor is appointed. “If the new director comes in and doesn’t like [our plan], then we sit down and think about where we’re going to go and how we’re going to get there based upon his or her vision of what the NTP should be doing,” Portier says. “But the advice we’ve gotten is still excellent advice, and it will help to chart whatever course we eventually take.”

## Figures and Tables

**Figure:**